# Exposure to synthetic hydraulic fracturing waste influences the mucosal bacterial community structure of the brook trout (*Salvelinus fontinalis*) epidermis

**DOI:** 10.3934/microbiol.2018.3.413

**Published:** 2018-06-11

**Authors:** Heather Galbraith, Deborah Iwanowicz, Daniel Spooner, Luke Iwanowicz, David Keller, Paula Zelanko, Cynthia Adams

**Affiliations:** 1 U.S. Geological Survey, Leetown Science Center, Northern Appalachian Research Laboratory, 176 Straight Run Road, Wellsboro, PA, USA; 2 U.S. Geological Survey, Leetown Science Center, National Fish Health Research Laboratory, 11649 Leetown Road, Kearneysville, WV, USA; 3 George Mason University, Department of Environmental Science and Policy, 4400 University Drive, Fairfax, VA, USA; 4 The Academy of Natural Sciences of Drexel University, 1900 Benjamin Franklin Pkwy, Philadelphia, PA, USA

**Keywords:** salmonid, mucus, disease, sublethal, bacteria, microbiome

## Abstract

Production of natural gas using unconventional technologies has risen as demand for alternative fuels has increased. Impacts on the environment from waste generated from these processes are largely unexplored. In particular, the outcomes of organismal exposure to hydraulic fracturing waste have not been rigorously evaluated. We evaluated the effects of exposure to surrogate hydraulic fracturing waste (HF waste) on mucosal bacterial community structure of the brook trout (*Salvelinus fontinalis*) epidermis. Brook trout are fish native to streams at risk to HF waste exposure. Here, fish were exposed to four treatments (control, 0.00%; low, 0.01%; medium, 0.10%; and high, 1.0% concentrations) of surrogate HF waste synthesized to mimic concentrations documented in the field. Epidermal mucus samples were collected and assessed 15 days post-exposure to determine if the associated bacterial community varied among treatments. We observed differences in epidermal mucosal bacterial community composition at multiple taxonomic scales among treatments. These community changes reflected compositional differences in taxa dominance and community similarity rather than losses or gains in taxonomic richness. The dominant bacterial genus that explained the greatest variation in community structure between exposed and unexposed fish was *Flavobacterium*. Two genera associated with salmonid diseases, *Flavobacterium* and *Pseudomonas*, were statistically more abundant in high treatments than controls. These results suggest that exposure to low levels of HF waste influences bacterial colonization and may lead to a disruption that favors bacterial populations associated with fish disease.

## 1. Introduction

Concern over greenhouse gas emissions, energy independence, and the need for cleaner combustible fuels has increased the demand for natural gas and the development of unconventional oil and gas (UOG) technologies [1]–[3]. Research to evaluate environmental impacts of these technologies has not kept pace with their rapid expansion and implementation. Hydraulic fracturing (HF) is one such technology whereby HF fluid containing water, sand and other proprietary mixtures (acids, friction reducers, surfactants, salt, scale inhibitors, pH-adjusting agents, iron control, corrosion inhibitors, and biocides), are pumped down the wellbore to fracture the underlying geologic formation [4],[5]. Water that returns to the surface (as either flowback or produced water, hereafter HF waste) also picks up constituents of the underlying rock formations including strontium (Sr), bromide (Br^−^), sodium (Na), calcium (Ca), barium (Ba) and chloride (Cl^−^) [1],[5].

Documented impacts of HF activities on surface waters are numerous, including toxicity (both lethal and sublethal) of accidental wastewater release, altered flow regimes from water withdrawal, and increased sedimentation due to drilling and road construction near streams and rivers [6]. HF waste has been linked to mortality events in resident aquatic biota, including documented fish and freshwater mussel kills [7]–[9]. Beyond direct mortality, HF development has also been documented to have impacts on fish, aquatic macroinvertebrate and microbial community structure, as well as tissue concentrations of heavy metals [10]–[13]. The underlying mechanisms leading to many of these effects have not been thoroughly investigated, but the combination of stressors (high salinity, heavy metals, and organics) may have both direct and indirect toxic effects on physiology and reproduction.

Many aquatic species including fish excrete an exopolymer matrix from goblet cells within the epidermis that hydrates rapidly upon contact with water to form considerable volumes of viscous mucus [14],[15]. This protective mucus acts as a first line of defense against a wide array of environmental contaminants, pathogens, parasites, and predators, but can also aid in buoyancy, swimming, communication and feeding [15],[16]. Despite the presence of a number of antibacterial factors within the epidermal mucus, bacteria inhabit this protective microenvironment as normal, healthy communities [14],[17]. In fact, under normal conditions these microbes themselves can provide further protections to the host against opportunistic bacterial pathogens. The ongoing colonization/extinction process of the epidermal mucus by microbes creates a biofilm. The resulting microbial community changes according to environmental flux such as location, temperature, osmolality and other conditions [14],[16],[18].

The goal of this study was to assess the effects of HF waste on the epidermal bacterial community of brook trout (*Salvelinus fontinalis*). Brook trout are native to streams throughout the Appalachian Basin, including the waters overlaying the Marcellus and Utica shale formations, which have recently experienced an increase in HF [19],[20]; however, suitable habitat has diminished due to invasive species introductions (brown and rainbow trout), and an intolerance to poor water quality and increased water temperatures [21],[22]. Few studies have addressed the direct or indirect effects of HF waste on brook trout (but see [9],[12],[13]), nor have the effects of HF waste on bacterial communities of the epidermal mucosal microhabitat been assessed. This study addresses the following objectives: (1) determine if epidermal bacterial community structure changes following exposure to surrogate HF waste; and (2) if affected, evaluate shifts in taxa that comprise bacteria pathogenic to salmonids in relation to simulated spill conditions.

## 2. Materials and methods

### 2.1. Exposure and sample collection

We collected brook trout fry (initial size range: 3.6–4.3 g each) from Big Brown Fish Hatchery (Effort, PA) in June 2014 and transported them to the USGS Northern Appalachian Research Laboratory (NARL) in Wellsboro, PA in aerated coolers. The fish were housed in the laboratory in flow-through fiberglass tanks (305 × 58 × 60.5 cm) containing approximately 920 L of well water, heated to reach 12 °C under a 12 h light and dark cycle. All fish were initially treated in a standing bath of 700 ppm oxytetracycline (OTC) for 6 hours upon arrival to the laboratory. Fish were then assigned and moved to treatment tanks and again treated with OTC prior to HF waste exposure. This OTC treatment was included for the specific task of labelling otoliths and was required for another component of a tandem research focus [23]. We utilized this opportunity to structure the experiment as a bacterial recolonization study. After secondary treatment, all fish were held in their respective groundwater-fed, flow-through tanks for 48 h prior to experimental exposure. When exposed (day 0), we converted each tank from flow-through to recirculating (all tanks with independent recirculating systems) and then dosed each tank with one of four surrogate HF waste solutions of varying intensity. We were unable to obtain authentic HF waste for this study; therefore, we synthesized a surrogate (hereafter “surrogate HF waste”) in the laboratory based on literature-reported chemical composition on day 5 for northcentral PA (Susquehanna, Potter, Bradford, Tioga, and Lycoming counties) reported by Hayes [24] (Table 1). To obtain desired dosing concentrations, the stock HF waste was serially diluted with distilled water (control, 0%; low, 0.01%; medium, 0.1%; and high 1.0%). All dosing dilutions comprised the same lot of distilled water freshly prepared the morning of the experiment to minimize contamination. Total dosing volumes ranged from 1–2 L reflecting 0.1–0.2% of each mesocosm respectively.

Each of the four treatment conditions (control, low, medium, and high) were run in duplicate with two tanks for each treatment and twelve fish in each tank (Table 1). Total starting fish biomass averaged (±SD) 47.0 (2.8) g yielding approximately 0.4 grams of fish per liter of water. Animal care was in accordance with USGS-approved animal care and use committee (IACUC) guidelines (approval date May 15, 2014). All tanks were aerated, the light-dark cycle maintained, and water temperature held constant at ∼12 °C by the use of external chillers and submersible heat exchangers. Tanks were cleaned to remove waste materials and mortalities every other day, and fish were fed twice daily a 50:50 mixture of Melick Aquafeed 1.5 mm pellets and BioVita Fry 2 mm pellets at 7.5% body weight while in holding and for the duration of the experiment. On day 2, we removed two fish from all tanks (as part of an accompanying study) and feed for each tank was adjusted accordingly. We semi-quantitatively analyzed nutrients on day 13 to assess waste accumulation (ammonium and nitrite) associated with tank re-circulation. Mortality was calculated per treatment.

**Table 1.** Chemical concentration (conc.) of three surrogate hydraulic fracturing treatments (low, medium, and high) used in a laboratory brook trout exposure study. Percentages are derived from literature-reported concentrations for north-central PA [24].

Compound	Low (0.01%) conc. (mg/L)	Medium (0.1%) conc. (mg/L)	High (1.0%) conc. (mg/L)
NaCl	4.8	48.4	479.1
KBr	0.1	0.6	6.4
CaSO_4_	0.0	0.1	0.5
BaCl_2_	0.5	5.4	53.2
CaCl_2_	1.2	11.8	117.1
FeCl_2_	0.0	0.1	1.1
MgCl_2_	0.1	10.8	10.6
SrCl_2_	0.2	2.2	21.3
LiCl	0.1	0.5	5.3
Mg_2_CO_3_	0.0	0.4	4.3
C_4_H_6_O_6_	0.0	0.2	1.5

On day 15, 5 fish from each tank (N = 40 total) were netted and euthanized with an overdose of tricaine methanesulfonate (MS-222) (Sigma Aldrich, St. Louis, MO). Epidermal mucus was then immediately collected from the midline of the lateral surface using a sterile inoculating loop and placed into a microcentrifuge tube containing DNA extraction buffer (200 µL TE: 10 mM Tris-HCl pH 8, 1 mM EDTA). Sample tubes of buffer and mucus were immediately placed on dry ice and then stored at −80 °C. Wastewater was transferred from all tanks to isolated holding drums and transported to an accredited HF fluid disposal facility (Bradford County Sanitation, Towanda, PA). During experimentation, all tank cleaning equipment (nets, brushes, siphons) were disinfected with a quaternary amine (Zep FS-amine Z, Zep®-Superior Solutions, Atlanta, GA) for at least 15 minutes and rinsed in clean well water prior to use in another tank.

### 2.2. Molecular analysis

Bacterial profiling of the mucosal community was performed at the National Fish Health Research Laboratory (NFHRL) according to Smith, Danilowicz and Meijer [25] with modification. In short, the TE buffered mucus sample was thawed and centrifuged at 15,700 RCF for 10 minutes. The buffer was then aspirated, leaving only the mucus pellet. Lysozyme (20 mg/mL) was added to the pellet and incubated for 30 minutes at 37 °C before extraction. We then extracted nucleic acids from the sample using the Qiagen DNeasy Blood and Tissue Kit (Valencia, CA) following the modification for gram positive bacteria.

Metagenomic amplicon sequencing libraries were prepared and sequenced according to 16S Metagenomic Sequencing Library Preparation (CT#: 15044223, Rev B) protocols established for the Illumina MiSeq System. The gene-specific primers used in this protocol target ∼460 bp of the V3 and V4 region of the 16S rRNA gene [26]. Final libraries were diluted 1:10 with nuclease-free (NF) water and quantified with the Qubit dsDNA HS Assay Kit (Thermo Fisher Scientific, Waltham, MA). DNA quality and amplicon size were determined using the Agilent DNA 1000 DNA kit (Santa Clara, CA). Pooled libraries were diluted to 4 nM using 10 mM Tris pH 8.5. A final 15 pM amplicon library was created with a 6.5% PhiX control spike and sequenced using the Illumina MiSeq 2 × 300 bp paired-end technology. All paired-end reads were uploaded to BaseSpace (Illumina). Taxonomic classification and read count metrics were determined using the 16S Metagenomics Application v.1.0. The algorithm used in this program is a high-performance implementation of the Ribosomal Database Project where reads reference an Illumina-curated version of the GreenGenes (May 2013) taxonomic database only [27]. The cut-off for assignment to OTUs was set at 97% sequence similarity. OTUs were accepted only if they consisted of at least 1% of all reads. Data used for this analysis were deposited in the National Center for Biotechnology Information BioProject database (PRJNA292446).

### 2.3. Statistical analysis

We used non-metric multidimensional scaling (NMDS) on Bray-Curtis scores followed by analysis of similarity (ANOSIM) to assess significant differences in bacterial community composition among treatments at three levels of taxonomic resolution (family, genus and operational taxonomic unit hereafter OTU). We followed ANOSIM with similarity percentages analysis (SIMPER) to identify community components contributing to the greatest dissimilarity among treatments. In addition to community similarity, we calculated richness and Shannon-Weaver diversity indices for each taxonomic level (family, genus, and OTU) to assess microbial biodiversity and compared the relative abundance (% of total OTU counts) of published salmonid disease causing genera among treatments. Data were tested to meet assumptions of normality and homogeneity of variances, and we used one-way ANOVA for each day followed by a Tukey honest significant difference (HSD) multiple comparison procedure to evaluate treatment-specific differences. All analysis were performed in R3.2.3 using the VEGAN and GGPLOT2 packages (cran.r-project.org).

## 3. Results

### 3.1. MiSeq analysis

**Figure 1.**
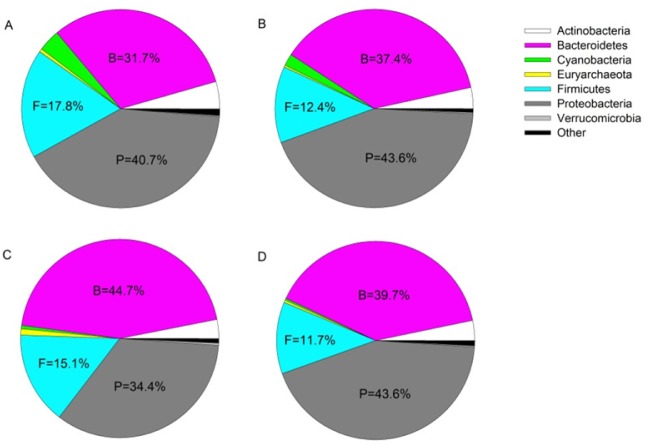
Relative sequence abundance (percentage of total classified) of bacterial phyla from experimental hydraulic fracturing waste treatment groups (control, A; low, B; medium, C; high, D). Percentages are reported for phyla with relative abundance greater than 5%. B: Bacteroidetes; F: Firmicutes; P: Proteobacteria.

**Figure 2.**
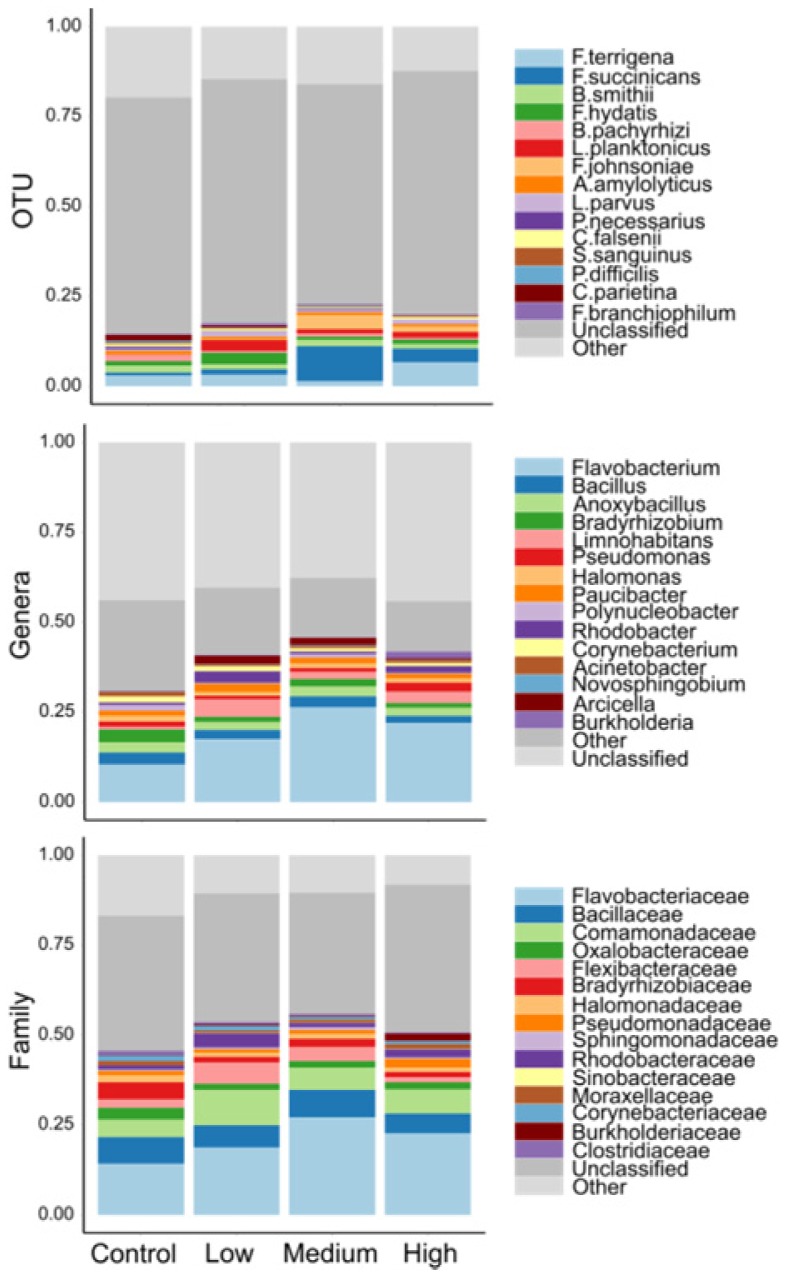
Mean relative abundances (percentage) of epidermal microbial taxa (OTU, genus, family) on brook trout exposed to four experimental hydraulic fracturing waste treatments (control, low, medium, and high).

After bioinformatic filtering of short and poor quality sequences, the amplicon sequencing resulted in 9.6 M reads (51.4% ≥ Q30 score and an error rate of 2.84%). Of these reads an average of 60.5% were classified to the taxonomic level of genus using the 16S Metagenomics Application. All of these sequences were successfully clustered in operational taxonomic units (OTU) with 97% identity, and were assigned to 821 genera distributed among 32 phyla. Individual OTUs were expressed as a percentage of the total summed OTUs across all samples. OTUs that comprised >0.01% of the total OTUs were retained and subsequently normalized to a 0–100% scale [28]. Relative abundance of taxonomic groups identified 4 dominant phyla of bacteria: Proteobacteria, Bacteroidetes, Firmicutes, and Actinobacteria. Over 75% of sequences identified represented the Proteobacteria or Bacteroidetes, with a slight shift toward Bacteroidetes in fish exposed to surrogate flow-back water compared to controls (Figure 1, Supplemental material A). At the genus level across all treatments, *Flavobacterium* reflected the greatest relative abundance (∼30%), followed by *Bacillus* (∼2.7%), and *Anoxybacillus* (∼2.6%) (Figure 2).

### 3.2. Sequence analysis

**Figure 3.**
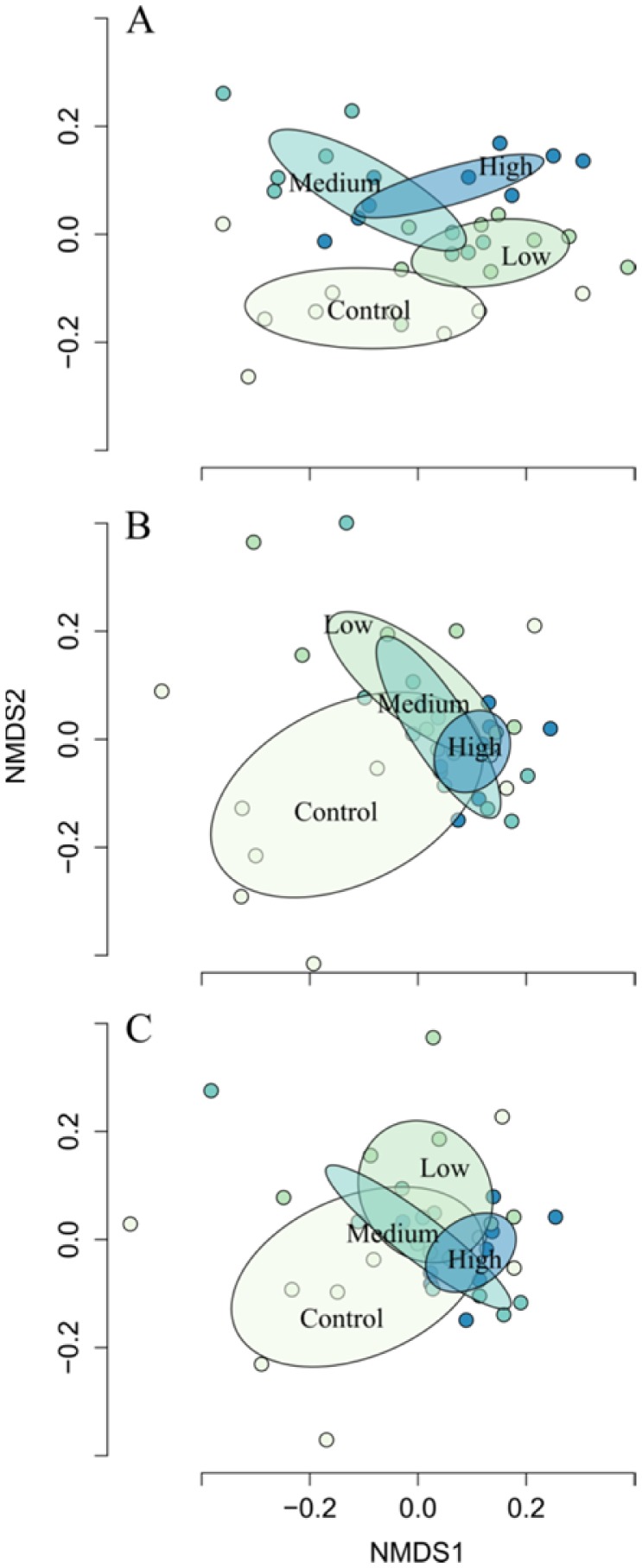
Non-metric multidimensional scaling plots of epidermal microbial community composition on brook trout exposed to four experimental hydraulic fracturing waste treatments (control, low, medium, and high). Plots are organized by taxonomic level (OTU, A; genus, B; and family, C). Circles represent treatment categories and ellipses 95% CI.

NMDS ordination converged on a solution with a stress of 0.16 (Figure 3). We found statistical significance for all ANOSIM analysis, with greater between-treatment variation in community dissimilarity relative to within-treatment variation (Table S1, Figure 3). ANOSIM R test statistics (difference of mean ranks between and within groups) generally increased with decreasing taxonomic resolution (Table S1). Moreover, we found epidermal bacterial community composition to be most variable on fish in control treatments, irrespective of taxonomic level of organization (Figure 3). The cumulative dissimilarity among treatments explained by community composition ranged from 10% (control-low, OTU level) to 62% (low-medium, family level) (Table 2).

**Table 2.** SIMPER analysis of the 5 (Rank) bacterial OTU's, genera, and families contributing the greatest dissimilarity among pairs of 4 hydraulic fracturing waste treatment groups (treatment). Percentage contributions are increasing in impact and cumulative.

Treatment	Rank	OTU	%	Genus	%	Family	%
Control-Low	1	*Flavobacterium terrigena*	0.10	*Flavobacterium*	0.23	*Flavobacteriaceae*	0.24
	2	*Limnohabitans planktonicus*	0.19	*Limnohabitans*	0.30	*Comamonadaceae*	0.34
	3	*Flavobacterium hydatis*	0.26	*Brandyrhizobium*	0.35	*Bacillaceae*	0.42
	4	*Calothrix parietina*	0.31	*Rhodobacter*	0.39	*Flexibacteraceae*	0.48
	5	*Candidatus Amoebophilus asiaticus*	0.36	*Arcicella*	0.43	*Oxalobacteraceae*	0.54
Control-Medium	1	*Flavobacterium succinicans*	0.18	*Flavobacterium*	0.30	*Flavobacteriaceae*	0.30
	2	*Flavobacterium terrigena*	0.26	*Bradyrhizobium*	0.35	*Comamonadaceae*	0.38
	3	*Flavobacterium johnsoniae*	0.33	*Limnohabitans*	0.38	*Bacillaceae*	0.46
	4	*Candidatus Amoebophilus asiaticus*	0.38	*Arcicella*	0.42	*Flexibacteraceae*	0.52
	5	*Calothrix parietina*	0.42	*Emticicia*	0.45	*Bradyrhizobiaceae*	0.57
Control-High	1	*Flavobacterium terrigena*	0.17	*Flavobacterium*	0.31	*Flavobacteriaceae*	0.31
	2	*Flavobacterium succinicans*	0.28	*Limnohabitans*	0.37	*Comamonadaceae*	0.39
	3	*Flavobacterium johnsoniae*	0.33	*Bradyrhizobium*	0.41	*Bacillaceae*	0.46
	4	*Limnohabitans planktonicus*	0.38	*Burkholderia*	0.44	*Bradyrhizobiaceae*	0.51
	5	*Candidatus Amoebophilus asiaticus*	0.42	*Pseudomonas*	0.48	*Oxalobacteraceae*	0.55
Low-Medium	1	*Flavobacterium succinicans*	0.18	*Flavobacterium*	0.27	*Flavobacteriaceae*	0.26
	2	*Flavobacterium terrigena*	0.26	*Limnohabitans*	0.35	*Comamonadaceae*	0.39
	3	*Flavobacterium johnsoniae*	0.33	*Rhodobacter*	0.40	*Flexibacteraceae*	0.48
	4	*Flavobacterium hydatis*	0.41	*Arcicella*	0.45	*Bacillaceae*	0.55
	5	*Limnohabitans planktonicus*	0.48	*Paucibacter*	0.49	*Rhodobacteraceae*	0.62
Low-High	1	*Flavobacterium terrigena*	0.18	*Flavobacterium*	0.26	*Flavobacteriaceae*	0.25
	2	*Flavobacterium succinicans*	0.29	*Limnohabitans*	0.32	*Comamonadaceae*	0.35
	3	*Limnohabitans planktonicus*	0.36	*Rhodobacter*	0.37	*Flexibacteraceae*	0.42
	4	*Flavobacterium hydatis*	0.42	*Pseudomonas*	0.42	*Bacillaceae*	0.49
	5	*Flavobacterium johnsoniae*	0.47	*Burkholderia*	0.46	*Rhodobacteraceae*	0.55
Medium-High	1	*Flavobacterium terrigena*	0.20	*Flavobacterium*	0.27	*Flavobacteriaceae*	0.27
	2	*Flavobacterium succinicans*	0.36	*Limnohabitans*	0.34	*Comamonadaceae*	0.39
	3	*Flavobacterium johnsoniae*	0.44	*Pseudomonas*	0.39	*Bacillaceae*	0.46
	4	*Limnohabitans planktonicus*	0.49	*Burkholderia*	0.44	*Flexibacteraceae*	0.53
	5	*Limnohabitans parvus*	0.52	*Arcicella*	0.48	*Burkholderiaceae*	0.57

**Figure 4.**
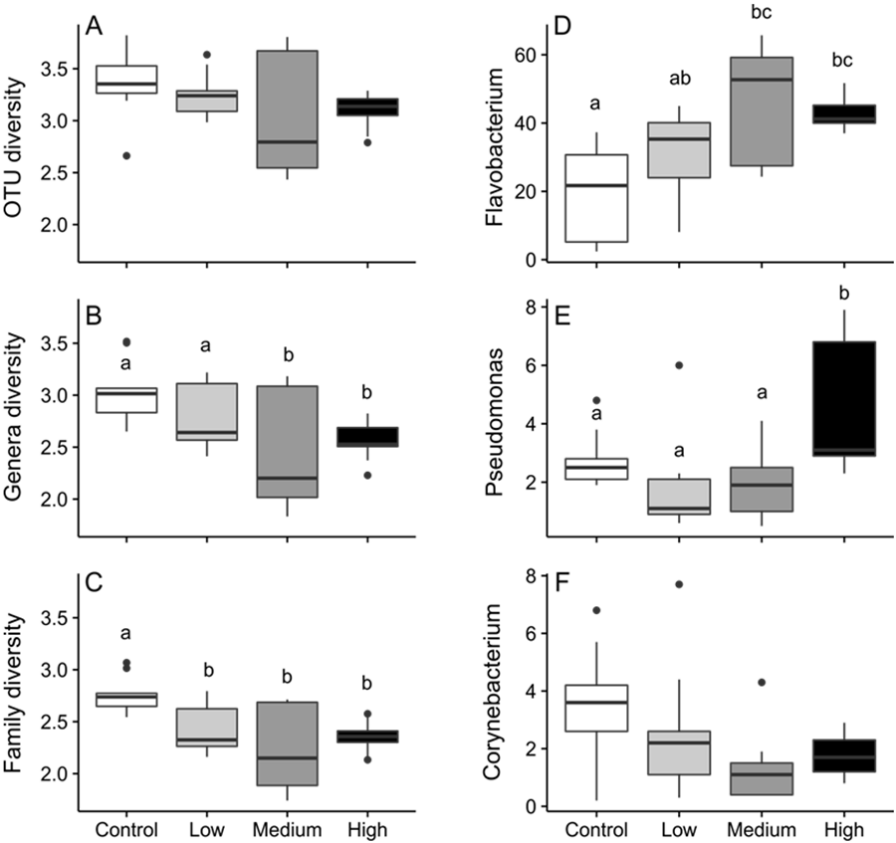
Box-and-whisker plots illustrating the taxonomic diversity (A–C; Shannon-Weaver Index) and the relative abundance of *Flavobacterium*, *Pseudomonas*, and *Corynebacterium* genera (D–F) for 4 experimental hydraulic fracturing waste treatments (control, low, medium and high). The horizontal line within each box represents the median diversity and dots represent outliers. Significant differences among treatments are denoted with different letters.

In light of the observed significant differences in community similarity, we found no significant differences in taxonomic richness with respect to treatment (Table S1, Figure S1). Shannon-Weaver diversity differed at the genus and family levels of taxonomic resolution, and most of these differences were among control and HF treatments (Table S1, Figure 4). According to the SIMPER analysis, *Flavobacterium* OTU's, explained the largest amount of variation among all treatment combinations (Table 2). *Flavobacterium* sp. followed by *Pseudomonas* sp. and *Corynebacterium* sp. were the three most prevalent genera associated with salmonid diseases (Figure 4). The relative abundance of *Flavobacterium* sp. was lowest in the control treatment and significantly differed from medium and high treatments (F_3,32_ = 3.48, *p* = 0.03). The relative abundance of *Pseudomonas* sp. was also lower in control compared to high treatments (F_3,32_ = 6.51, *p* = 0.001) (Figure 4). Conversely, the relative abundance of *Corynebacterium* sp. appeared highest in the control treatments, yet the pattern was not significant (F_3,32_ = 2.64, *p* = 0.07) (Figure 4). Ambient nutrient concentrations generally increased when the tanks were converted from flow-through to re-circulating. Moreover, while nitrite was generally consistent among treatments, ammonium concentrations were elevated in the high HF treatments (Table S2). No mortality was observed in treatment or control tanks during the 15 days of exposure.

## 4. Conclusions

The results of this study suggest that the reestablishment of mucosal epidermal bacterial community composition is impacted by exposure to low-level surrogate HF waste, with differences evident at multiple taxonomic scales. Mucosal microbial communities can provide a first line of protection against infection [22],[29],[30]. Our results suggest that exposure to HF water, could compromise the community composition of this protective layer: while not directly lethal at these levels, HF waste resulted in higher abundance of potentially pathogenic bacteria in exposed versus unexposed fish. While it is difficult to directly compare documented spills to the conditions reported in this study as resulting water chemistry is influenced by HF water composition (which can be proprietary), stream size, and discharge, the concentrations of Cl^−^, the primary component in our HF water, is on par with those documented in recent spills. Salinity (converted from conductivity, assuming a water temperature of 25 °C) following spills in Acorn and Dunkard Creeks (KY and WV/PA, respectively) ranged from a low of 116 mg/L to nearly 23,000 mg/L [7],[31]. Our high level treatments were on par with the lower end of reported spill concentrations in these creeks, suggesting that our results are likely a conservative estimate of what may occur under more extreme spills.

Fish mucosal microbiomes have been shown to change following experimental exposure to stressors. Xia et al. [32] demonstrated altered seabass intestinal microbiomes in response to starvation, with dramatic increases in Bacteroidetes, the second most dominant group in our studies, and loss of Betaproteobacteria. Hess et al. [33] documented differences in clownfish gill microbial communities following exposure to suspended sediment, with novel bacterial taxa and increased pathogenic OTUs (*Flavobacterium*, *Pasteurella*, *Edwardsiella*, and *Chryseobacterium* spp.) in treatment groups. In our study, the number of taxa (richness) did not differ among treatments at any level of taxonomic organization. Instead, we found that community similarity and taxonomic diversity differed with respect to treatments, suggesting that the relative dominance of particular taxa within the community shifted with respect to our treatments. Similar to Hess [33], we observed increased prevalence of two well-documented disease causing genera suggesting that prolonged exposure to certain levels of surrogate HF waste may put fish at higher risk of infection, or the potentially synergistic effects of chemical toxicity and disease. Of particular interest in our findings is the genus *Flavobacterium*, which is considered to be ubiquitous in temperate freshwater and known to include several pathogenic species (including those causing coldwater disease, rainbow trout fry syndrome, columnaris, and bacterial gill disease [34]–[36]). Given the increase in this genus in ours and other studies after exposure to stressors [33], it suggests that *Flavobacterium* OTUs are able to capitalize on some change in conditions, providing an enhanced environment for this pathogenic bacterial species.

Because tanks in our study were fed from the same source water and because bacteria were presumably ubiquitous within our laboratory, it is reasonable to assume that the bacterial communities on these fish and in the tanks (water column and tank walls) comprised similar taxonomic composition at the onset of the simulated HF waste exposure [37],[38]. As such, we envision three, non-mutually exclusive scenarios for which taxonomic composition may have been altered: (1) colonization by new taxa; (2) mortality or local extirpation of particular taxa; and (3) a shift in relative dominance of particular taxa. Because tanks were randomized and in close proximity to one another, it is unlikely that treatment-specific colonization of novel taxa occurred (i.e. scenario 1) [38] (although sterilization of the inoculum HF water prior to addition, not done in this study, would have reduced the likelihood of a novel species introduction). It is more likely that changes in environmental conditions associated with the treatments selected for or against bacterial taxa with specific traits (scenarios 2 and 3) [38]. Local extirpation of certain taxa may have been a function of either direct mortality due to the antimicrobial effects of salt or other HF water components (e.g., heavy metals; scenario 2) or indirect effects such as an altered competitive arena in which certain bacteria had competitive advantages over others (scenario 3).

The mechanism(s) by which the competitive arena may influence bacterial communities (scenario 3) in response to HF water is unclear. Mucosal bacterial communities may simply respond to changes in water column environmental conditions, with little to no interaction with their host fish [39]. For example, Wu, Wang, Angert et al. [39] examined gut microbial communities of grass carp and found that while the community was dominated by taxa with traits specific to digestion, there was a strong similarity to water and sediment communities. However, fish mucous has been shown to provide a novel niche for mucosal bacterial evolution [40]. Alternatively, fish exposure to surrogate HF waste could have differentially impacted antimicrobial peptide and protein production within the mucus, or ammonium excretion from the nearby gills, leading to fish-mediated differences in bacterial community structure [16],[41]. Along these same lines, the ability of bacteria to invade the mucus layer, differential rates of mucus production (and thereby sloughing of bacteria), physical properties of mucus, and OTC exposure all may have caused differences in turnover time of bacterial taxa colonizing and recolonizing the epidermis from the water [41],[42]. Future studies combining microbiome evaluations with host mucosal functional immune response could help to shed light on which, if any, of these mechanisms could be contributing to these results [32].

Recent field studies have addressed shifts in stream microbial community composition in response to energy development. Trexler et al. [11] evaluated water, sediment, and biofilm microbial community structure (OTU) among sites impacted by natural gas extraction activities. They documented decreases in community metrics (species richness, evenness, and diversity) in areas with natural gas production along with correlations between pH and richness with corresponding shifts in dominance towards taxa suited to low pH or high methanotrophic environments. The pH of HF water varies with time after recovery and location of extraction sites and was not a major component evaluated in our study [24]. However, given the strong correlations between field microbial communities and pH [11], laboratory evaluation of its effects on mucosal microbiomes is warranted. Similarly, studies comparing the microbial community composition of hatchery-reared trout to those of native trout as well as the effects of HF exposure on native mucosal microbial community composition are critical. Hatchery-reared fish provide greater replication and control over age and physiological history of experimental animals. However, the applicability of this study to wild populations should be evaluated.

Mucosal microbial community composition is apparently variable even among hatchery-reared populations. Boutin, Bernatchez, Audet et al. [37] identified dominant bacterial taxa in apparently healthy hatchery-reared brook trout. While there were notable similarities with our study (e.g. Proteobacteria were the dominant phyla), Bacteroidetes were the second most dominant phyla in our study as opposed to Actinobacteria in Boutin, Bernatchez, Audet et al. [37]. Differences in bacterial communities between these studies could have been due to experimental location, system design, environmental conditions (e.g., water temperature), fish developmental stage, and source water (well vs. UV-sterilized water). Experimental requirements for a simultaneously-conducted study [23] required the pre-treatment of our fish with OTC for otolith marking. This likely influenced the context of our results in that we quantified recolonization of bacteria in response to HF waste. Therefore, while our treatments presumably began with similar taxonomic pools, they likely differed from the initial pools characterized in Boutin, Bernatchez, Audet et al. [37] (that did not use OTC), complicating cross study comparisons. Follow-up studies that manipulate, standardize, and characterize initial species composition in experiments are needed. This initial composition combined with environmental conditions, likely play a role in driving stressor-mediated community shifts.

Advancements in high throughput sequencing have greatly facilitated evaluation of bacterial communities of vertebrates. Data gleaned from human gut microbial communities suggest that critical assemblages of microbes, in part, define a healthy or unhealthy host [43]. Similar findings have resulted from studies of surface flora in a variety of organisms including fishes, where it has been suggested that microbial communities confer a benefit to their host in part by providing protection against opportunistic infections [22],[29],[30]. Our results suggest that low-level exposure to HF waste can impact the recolonization of the natural epidermal bacterial community of fish that may be critical for maintaining fish health. Further assessments that quantitatively isolate disease-causing bacterial species in exposed and unexposed fish and document fish histology in response to chronic low-level HF waste exposure would provide a useful follow-up to facilitate a mechanistic understanding of fish susceptibility to HF waste. Additionally, expanding this work to include more sampling points over a longer time period may lend insight into the resiliency and recovery response of bacterial communities to HF waste, identify the time frame during which brook trout are susceptible to disease following an HF waste spill, and serve as a bioindicator of water quality in the future [44],[45].
