# Supplementary material for: Exposure to synthetic hydraulic fracturing waste influences the mucosal bacterial community structure of the brook trout (Salvelinus fontinalis) epidermis
Source: AIMS Microbiol. 2018 Jun 11;4(3):413–27. doi: 10.3934/microbiol.2018.3.413 (PMC6604949; doi:10.3934/microbiol.2018.3.413)
Supplement: Supplementary file 1 [file microbiol-04-03-413-s1.pdf]

Microbiology

AIMS Microbiology, 4(3): 413–427.
DOI: 10.3934/microbiol.2018.3.413
Received: 08 January 2018
Accepted: 16 May 2018
Published: 11 June 2018

http://www.aimspress.com/journal/microbiology

Research article

Exposure to synthetic hydraulic fracturing waste influences the mucosal bacterial community structure of the brook trout (*Salvelinus fontinalis*) epidermis

Heather Galbraith[1,]*, Deborah Iwanowicz[2], Daniel Spooner[1,3], Luke Iwanowicz[2], David Keller[4], Paula Zelanko[3] and Cynthia Adams[2]

[1] U.S. Geological Survey, Leetown Science Center, Northern Appalachian Research Laboratory, 176 Straight Run Road, Wellsboro, PA, USA
[2] U.S. Geological Survey, Leetown Science Center, National Fish Health Research Laboratory, 11649 Leetown Road, Kearneysville, WV, USA
[3] George Mason University, Department of Environmental Science and Policy, 4400 University Drive, Fairfax, VA, USA
[4] The Academy of Natural Sciences of Drexel University, 1900 Benjamin Franklin Pkwy, Philadelphia, PA, USA

* **Correspondence:** Email: hgalbraith@usgs.gov; Tel: +5707243322230; Fax: +5707242525.

Supplementary

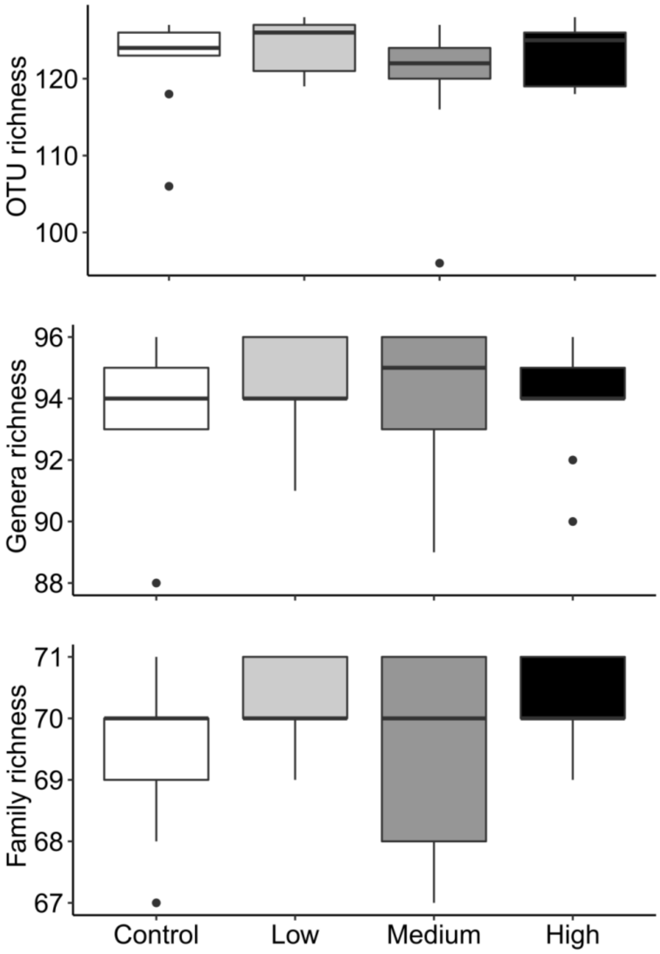

Figure S1. Box and whisker plots illustrating taxonomic richness for 4 experimental hydraulic fracturing waste treatments (control, low, medium and high). Note differences in y-axis scales.

Table S1. Statistical output comparing brook trout epidermal bacterial community structure (Analysis of Similarity, ANOSIM; Community Richness ANOVA; and Shannon-Weaver Diversity ANOVA) across 4 experimental hydraulic fracturing waste treatments (control, low, medium, and high) and at three taxonomic levels (OTUs, genus, and family). Bold and italicized text represents significant differences among treatments.

Community metric	Day	OTU Test statistic	P-value	Genus Test statistic	P-value	Family Test statistic	P-value
Analysis of Similarity (ANOSIM)	15	***R = 0.28***	***0.001***	***R = 0.17***	***0.001***	***R = 0.12***	***0.002***
Richness (ANOVA)	15	$F_{3,32} = 1.06$	0.38	$F_{3,32} = 0.27$	0.84	$F_{3,32} = 0.99$	0.41
Shannon-Weaver Diversity (ANOVA)	15	$F_{3,32} = 1.21$	0.32	***$F_{3,32} = 4.17$***	***0.01***	***$F_{3,32} = 6.29$***	***0.001***

Table S2. Ranges of ammonium and nitrite concentrations in 4 hydraulic fracturing waste treatments and the flow-through holding tank on day 13 of experimental exposure.

Treatment	Ammonium (mg NH_{4+})	Nitrite (mg NO_2^-)
Holding tank (flow through)	<0.2	<0.05
Control	0.4–1.5	0.5–0.8
Low	0.8–1.5	0.2–0.8
Medium	0.8–1.0	>0.8
High	1.5–2.0	0.3–0.8

Supplemental Material A (microbiol-04-03-413-s2): Relative sequence abundance of bacterial taxa (OTUs) from 4 HF waste exposure treatments (control, low, medium, and high) implemented by Krona software [1] (HTML files,). See Table 1 for specific treatment concentrations.

References

1. Ondov BD, Bergman NH, Phillippy AM (2011) Interactive metagenomic visualization in a Web browser. *BMC Bioinformatics* 12: 385.

AIMS Press

© 2018 the Author(s), licensee AIMS Press. This is an open access article distributed under the terms of the Creative Commons Attribution License (http://creativecommons.org/licenses/by/4.0)
